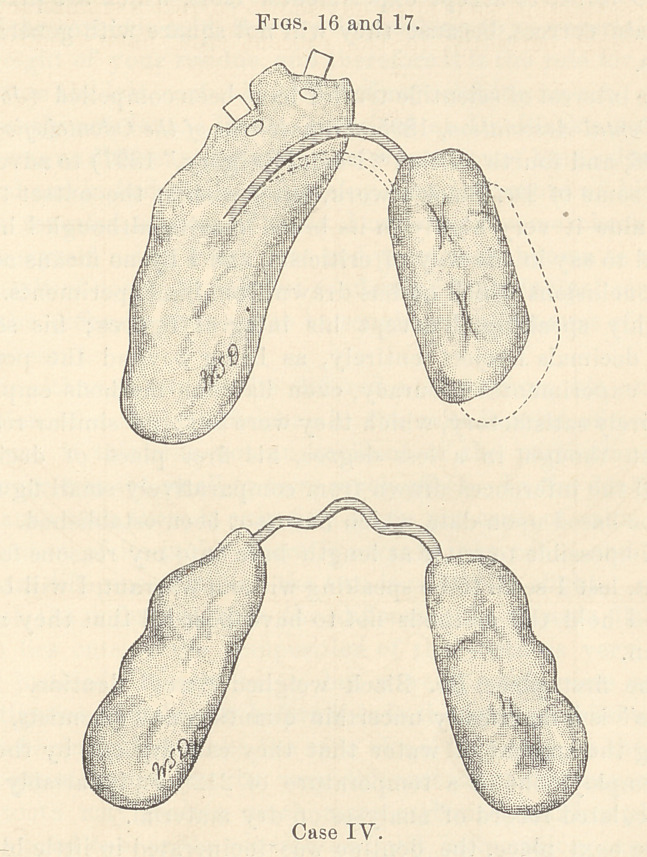# Regulating without Extraction *Versus* Extraction for Regulating; Some Typical Comparative Results

**Published:** 1897-12

**Authors:** Wm. Slocum Davenport

**Affiliations:** Paris, France


### International Dental Journal.

Vol. XVIII. December, 1897. No. 12.

### Original Communications.[1]

### REGULATING WITHOUT EXTRACTION *VERSUS* EXTRACTION FOR REGULATING; SOME TYPICAL COMPARATIVE RESULTS.

BY WM. SLOCUM DAVENPORT, D.D S., PARIS, FRANCE.

(Concluded from page 631.)

Case IV.—Figs. 10, 12, and 14 represent the mouth of a girl fourteen years of age, whose four sixth-year molars had been extracted by her former dentist before the eruption of the twelfth-year molars. Figs. 11, 13, and 15 show the position of the teeth after treatment. When the twelfth-year molars erupted the forward and inner tipping was so great that the retaining power of the cusps was lost.

The lower jaw was forced to the left five millimetres, as is shown in Fig. 10, Line *A*.

Line *B*, Fig. 10, shows the faulty articulation of the teeth on the left side. The lower teeth articulate the width of a bicuspid too far backward, while the upper teeth of this side fall within the lower arch.

[1] The editor and publishers are not responsible for the views of authors of papers published in this department, nor for any claim to novelty, or otherwise, that may be made by them. No papers will be received for this department that have appeared in any other journal published in the country.

Line *A*, Fig. 11, shows the medium line in correct relation above and below after treatment. The articulation of the right side was comparatively perfect at first.

Figs. 10 and 11.

Case IV.

Fig. 16 illustrates the plate used to force the bicuspids and molars of the left side backward, and at the same time spread the arch. The plate was inserted with the springs in such a position that the force was brought to bear in the direction indicated by the dotted line.

Fig. 17 illustrates the plate used to continue the spreading of the back teeth, including the canines. A similar plate was used to spread the lower arch in conformity with the upper.

A comparison of Line *A*, Figs. 10, 11, 12, and 13, shows the distance the lower arch moved forward and to the right, thus correcting the irregularity.

Figs. 12 and 13.

Case IV.

Line *C*, Figs. 12, 13, 14, and 15, shows how much the upper teeth on the left side were moved backward.

Comparison of Figs. 14 and 15 shows the changes which took place in the upper arch.

Case IV. is instructive in that it shows the bad position into which the teeth have fallen after extraction of the first molars. Further extraction, as is often advised to correct a similar acquired defect, would certainly, in the present case, have resulted dis-

Figs. 14 and 15.

Case IV.

astrously. But by the plan of regulating without further extraction the normal relation of the arches to each other and to the true median line were restored; and the articulation also, as far as possible, with the first molars absent.

This seems to be a common form of acquired irregularity entirely due to extraction of teeth, especially when practised (as is often done) with the idea of correcting what is considered to be an

Figs. 16 and 17.

Case IV.

overcrowded arch. I have yet to find an arch of this kind that could not be expanded to properly accommodate the teeth.

## Figures and Tables

**Figs. 10 and 11. f1:**
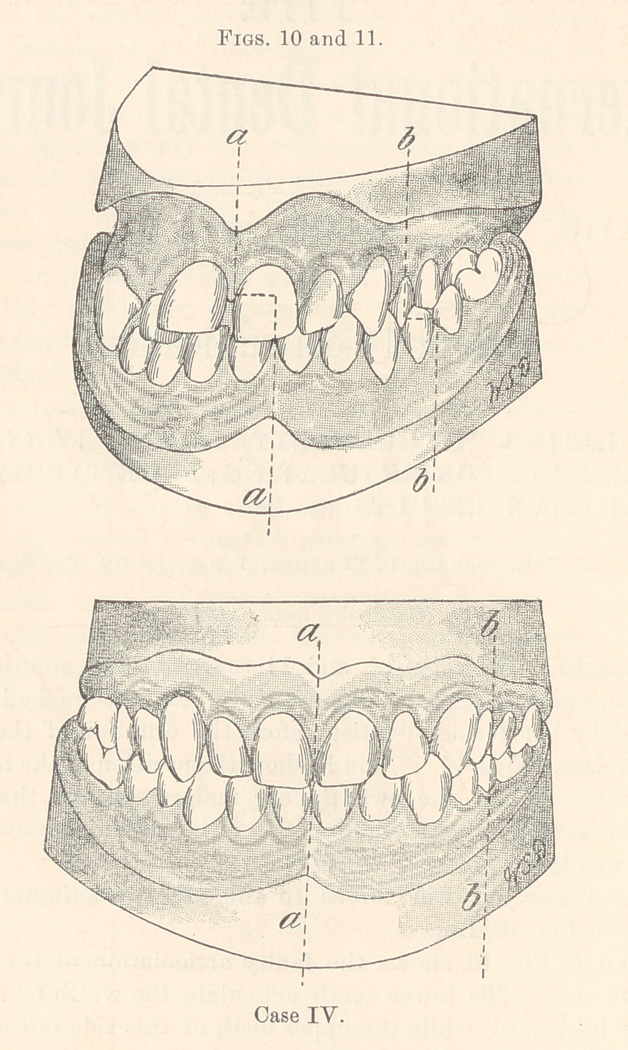


**Figs. 12 and 13. f2:**
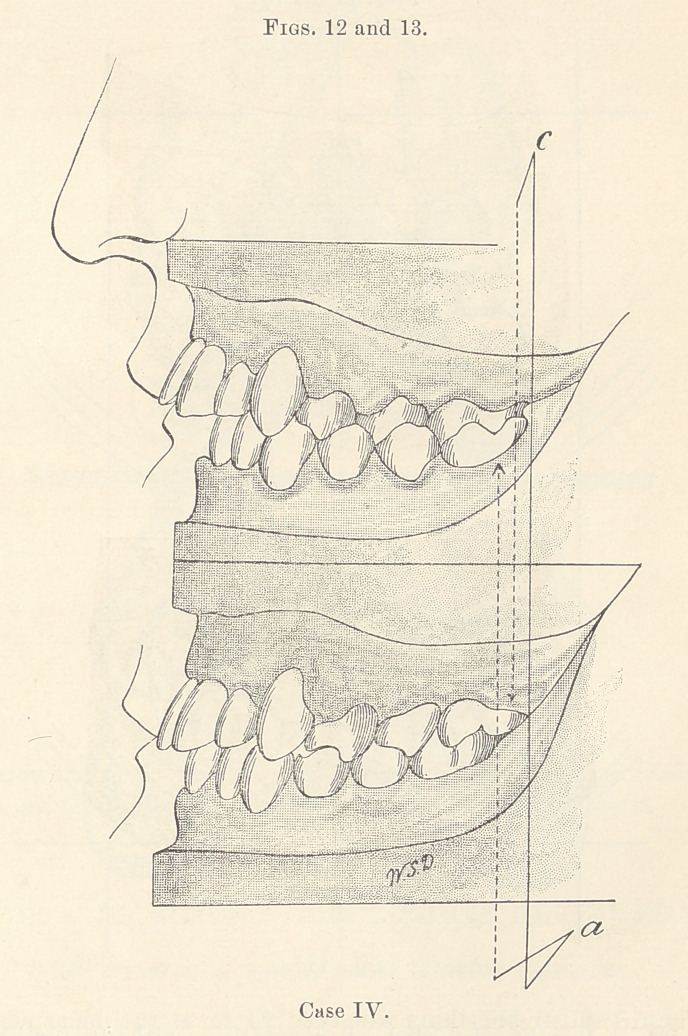


**Figs. 14 and 15. f3:**
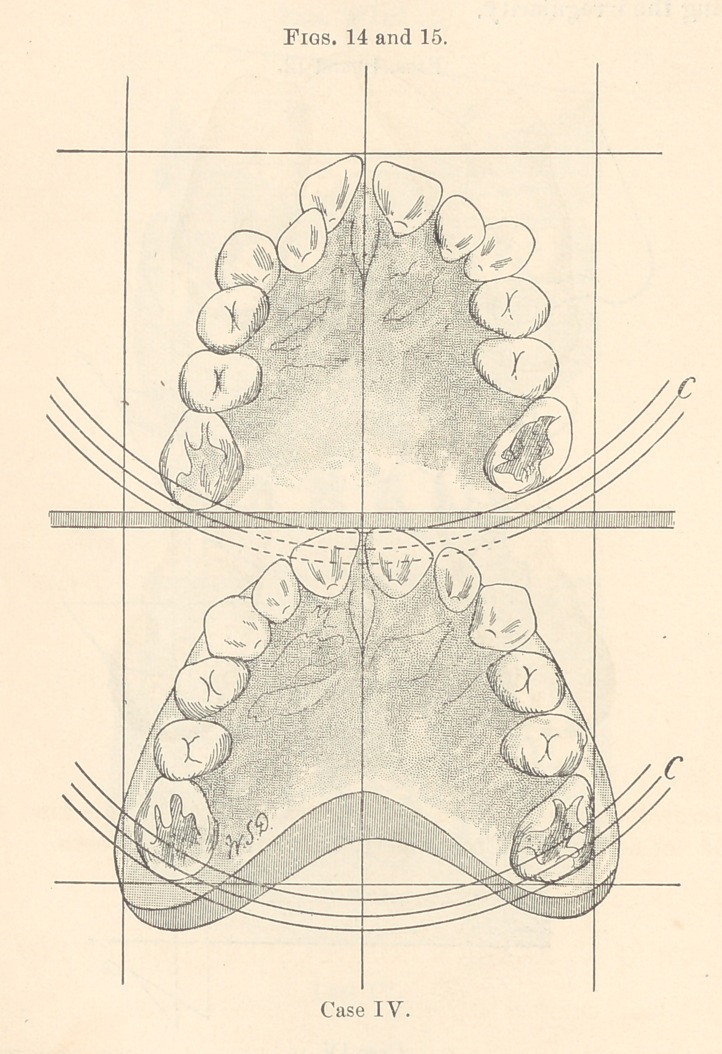


**Figs. 16 and 17. f4:**